# Self-beneficial transactional social dynamics for cooperation in Shwachman-Diamond syndrome: a mixed-subject analysis using computational pragmatics

**DOI:** 10.3389/fpsyg.2024.1459549

**Published:** 2025-01-22

**Authors:** Arthur Trognon, Natacha Stortini, Coralie Duman, Nami Koïdé, Ewa Skupinska, Hamza Altakroury, Alizée Poli, Loann Mahdar-Recorbet, Blandine Beaupain, Jean Donadieu, Michel Musiol

**Affiliations:** ^1^CLINICOG, Nancy, France; ^2^CNRS, ATILF, Lorraine University, Nancy, France; ^3^Campus Lettres et Sciences Humaines, Lorraine University, Nancy, France; ^4^Faculté de Psychologie, Strasbourg University, Strasbourg, France; ^5^Epitech, Lyon, France; ^6^French Reference Center for Langerhans Cell Histiocytosis, Trousseau Hospital, AP-HP, Paris, France; ^7^Institut National de Recherche en Informatique et en Automatique (INRIA), Nancy, France

**Keywords:** Shwachman-Diamond syndrome, computational psychology, mathematical psychology, psycholinguistics, interactive psychology, pragmatics, deep-learning

## Abstract

**Background:**

Shwachman-Diamond Syndrome (SDS) is a rare genetic disorder with documented cognitive and behavioral challenges. However, its socio-pragmatic dynamics remain underexplored, particularly in cooperative interactions where social norms and economic considerations intersect.

**Objective:**

This study investigates the socio-behavioral dynamics of SDS, focusing on how children with the condition navigate cooperative interactions. Using computational pragmatics, we aimed to identify the underlying principles guiding their social behavior.

**Methods:**

A cohort of 10 children (5 SDS, 5 matched controls) participated in ecological and cognitive tasks, including the WISC-V “Comprehension” subtest, NEPSY-II social perception tasks, and the Trognon Ecological Side Task for the Assessment of Speech-Act Processing (TEST-ASAP). Dialogues were analyzed using the Topological and Kinetic (2TK) model and a Recurrent Neural Network (RNN), enabling fine-grained computational insights into their interaction patterns.

**Results:**

Children with SDS exhibited cooperative behaviors shaped by perceived economic benefits, often at the expense of established social norms. Unlike behaviors classically observed in other pathologies such as autism spectrum disorders, where responses are influenced by the directness of communication, SDS behaviors were driven by personal gain, regardless of the indirectness of requests. Computational analyses revealed strong divergences in dialogical alignment when tasks lacked direct benefits, even with corrective prompts.

**Conclusion:**

SDS children demonstrate a transactional approach to social interactions, prioritizing personal benefits over cooperative norms. Using our unique dialogic and computational frameworks, we show that perceived personal gain strongly shapes their cooperation patterns. These findings underscore the need for targeted interventions to enhance pragmatic skills and adaptive functioning in SDS, given their unique interaction profiles.

## 1 Introduction

Shwachman-Diamond syndrome (SDS), a rare autosomal recessive disorder, occurs in approximately one in 75,000 live births (Oyarbide et al., 2016), though actual incidence may be higher due to its phenotypic variability and diagnostic challenges (Frattini et al., 2021; Oyarbide et al., 2016). The disorder is primarily caused by biallelic inactivation of the SBDS gene, located at the 7q11.21 locus of chromosome 7, which is expressed in nearly all adult tissues, including the myocardium and central nervous system (Boocock et al., 2003). In humans, the most frequent mutations of the SBDS gene result in neutropenia, exocrine pancreatic insufficiency, bone marrow failure, and skeletal dysplasia (Aggett et al., 1979; Ginzberg et al., 1999; Mack et al., 1996). Other complications such as developmental delays or neuropsychiatric difficulties (Perobelli et al., 2012, 2015), anemia (Dror et al., 2011), hepatic cytolysis (Cipolli et al., 1999), cardiac pathologies (Fleitz et al., 2002), and type I diabetes (Gana et al., 2011) are also observed. Additionally, patients have an increased risk of developing myelodysplastic syndrome and acute myeloid leukemia, occurring in 19 to 36% of cases (Aalbers et al., 2013). National registries provide specific data, with Italian and French registries reporting annual incidences of approximately 3 (Minelli et al., 2012) and 4.4 cases per year, respectively. In France, the prevalence of SDS is estimated at 4.2 cases per million people (Donadieu et al., 2020).

However, while numerous studies document the organic effects, there are very few studies that have investigated the neuroanatomical and behavioral specifics in this clinical condition.

One specific study (Perobelli et al., 2015) described the impact of SBDS mutations on nervous system development. Data suggested notable differences in cortical thickness predominantly in the temporal and parietal lobes, with the most pronounced thickening observed in the left limbic-anterior cingulate cortex. Contrarily, areas within Broca’s region in the left hemisphere exhibit a reduced thickness compared to controls, suggesting a potential regional specificity in brain morphology alterations in SDS. Further, functional MRI (fMRI) measure employing the Stroop task reveal distinct patterns of brain activation in SDS patients compared to controls. Specifically, during this cognitive task, SDS patients show reduced brain activity in key areas including the middle left frontal gyrus, left precuneus, and left hippocampus. Conversely, control subjects exhibit more pronounced activation in the anterior cingulate cortex, orbital cortex, and left inferior frontal gyrus. In other hands, Diffusion-Tensor Imaging and Tract-Based Spatial Statistics analyses revealed widespread disruptions in white matter pathways, with increased fractional anisotropy in the fronto-callosal area, the right frontal-external capsulae pathway, the left fronto-parietal region, the right pontine region and in the anterior medial temporal lobes, including pathways associated with the limbic system.

These neuroimaging findings thus offer a compelling neurobiological framework for understanding the observed cognitive impairments observed in Shwachman-Diamond Syndrome. Since the first observation of the pathology, studies have consistently reported that individuals with Shwachman-Diamond Syndrome display a range of cognitive deficits, including reduced intellectual efficiency and dysexecutive syndrome (Aggett et al., 1979; Cipolli et al., 1999; Kent et al., 1990; Kerr et al., 2010; Perobelli et al., 2015), with significant variability within the patient group (Perobelli et al., 2012).

Despite these insights, the literature still lacks detailed exploration into how these cognitive dysfunctions may translate into everyday social interactions, particularly in complex exchanges such as dialogic interactions.

In recent years, during discussions held at the Annual Meetings of the French National Registry for Chronic Neutropenias, parents of children with Shwachman-Diamond Syndrome have overwhelmingly reported difficulties in achieving cooperation (i.e., the coordinated efforts of two or more individuals working toward a shared goal; Sebanz et al., 2006; Tomasello, 2009) from their children. They almost unanimously cited homework and other tedious tasks as problematic. To elicit cooperation on these challenging or unpleasant tasks, parents concurred that offering a short-term reward was an effective strategy.

This consistent observation from parents could thus provide a valuable framework for analyzing the interactive behaviors of children with Shwachman-Diamond and further guide targeted interventions to improve patient cooperation and daily functioning.

In this study, we explored the interactive behaviors of children with Shwachman-Diamond Syndrome using dialogical computational modeling. We employed two distinct tasks: the Wechsler Intelligence Scale for Children (WISC-V; Wechsler, 2016) as a model of homework realization in clinical settings, and a specifically designed experimental tool, the Trognon Ecological Side Task for the Assessment of Speech-Act Processing (TEST-ASAP; derived from Gerardin-Collet, 1999). This task, integrated into the neuropsychological evaluation process to ensure its ecological validity, is subdivided into three subtasks that measure inferential aspects, behavioral induction under instruction without benefit, and behavioral induction requiring prior inference but carrying benefits. These subdivisions aim to highlight dissociations in behavioral responses. Motivated by parental reports that tangible rewards are necessary to enhance cooperation, we hypothesized that perceived economic incentives significantly influence SDS children’s social interactions. Thus, the TEST-ASAP was developed to distinguish between the costs of interactions and the directiveness of given instructions, allowing us to examine the impact of incentives on cooperation and communicative behavior in these children.

Dialogical data for these tasks were recorded, transcribed verbatim, and then analyzed using Trognon’s Topological and Kinetic (2TK; Trognon, 2022) model, a mathematical analytical framework for unifying the three dominant approaches to interactive analysis: Theoretical Linguistics and Ludics (Girard, 1987, 2001; Lecomte and Quatrini, 2011), Computational Linguistics and Segmented Discourse Representation Theory (SDRT; Asher and Lascarides, 2003; Caelen, 2003; Caelen and Xuereb, 2014, 2017), and Interlocutionary Analysis (Trognon, 1992). The joint use of human-computer interaction and human-human interaction analysis techniques to study natural dialogue is particularly justified here by the construction of the task, whose outcome and course are predictable in the neurotypical subject (i.e., ideal dialogue having the characteristics of a cooperative dialogue where the interactants help each other toward a common goal), and of which all other configurations would be considered as artifactual (i.e., non-optimal and detected by these methods).

In the present article, we will present the data gathered from the TEST-ASAP for a set of five children from the SDS cohort, in comparison with control subjects matched in age, sex, and socio-cultural level. Furthermore, we will detail the interactions of two patients from the French SDS cohort: an 8-year-old girl during the administration of the “Comprehension” subtask from WISC-V and an 11-year-old boy for which we compared dialogical metrics during three situations: a cooperative task from the TEST-ASAP; a cognitive task from the WISC-V; and a free speech situation. These cases are particularly compelling as neither child displays an intellectual disability nor encounters scholastic challenges. Yet, their parents have indicated significant issues in securing their cooperation.

## 2 Materials and methods

### 2.1 Study design

The study involved two participant groups: children and adolescents aged 7–17 years and 11 months with genetically confirmed Shwachman-Diamond Syndrome (SDS; Group A, *n* = 5) and matched controls without pathology (Group B, *n* = 5). Participants in the SDS groups were recruited through the French National Registry for Chronic Neutropenias, while controls were recruited through advertisements in general medical practices. Recruitment criteria ensured age, sex, and socio-economic status matching across groups. Exclusion criteria included sensory or linguistic impairments interfering with task performance, history of significant head trauma, inability to consent, or participation in concurrent studies.

All child participants underwent a structured assessment protocol at Hôpital Armand Trousseau (Paris). The protocol was conducted over two half-days, totaling 3.5 h of testing with a 2-h lunch break. The first half-day included an anamnesis interview (30 min), administration of the Wechsler Intelligence Scale for Children–Fifth Edition (WISC-V; 90 min), and the Trognon Ecological Side Task for the Assessment of Speech-Act Processing (TEST-ASAP; 10 min). The second half-day featured the NEPSY-II neuropsychological assessment (60 min), focusing on social perception. After testing, a preliminary oral feedback session was held with parents, including a preliminary review of performances and an opportunity to address questions.

### 2.2 Subjects

Ten (*n* = 10) not preselected children (SDS: *n* = 5; Controls: *n* = 5) from the French population participated in this study. Ethical informed written consents were obtained from parents with their children’s consent, in agreement with the Declaration of Helsinki. The study was approved by the “Comité de Protection des Personnes Sud-Est VI.” Full measures were available for all subjects.

### 2.3 Cognitive and neuropsychological assessment

Table 1 presents an overview of the tests from these batteries, along with brief descriptions of the constructs they measure. To ensure a comprehensive assessment of cognitive and socio-cognitive functions, we employed two standardized batteries: the Wechsler Intelligence Scale for Children–Fifth Edition and the NEPSY-II.

**Table 1:** Summary of neuropsychological tests employed.

Battery	Test	Description
WISC-V	Similarities	Identifies the commonalities between two objects or concepts. Contributes to the Verbal Comprehension Index (VCI).
WISC-V	Vocabulary	Requires naming and defining words (visual and auditory prompts). Contributes to the VCI.
WISC-V	Block design	Involves assembling bi-colored blocks to replicate a visual model under timed conditions. Contributes to the Visual Spatial Index (VSI).
WISC-V	Matrix reasoning	Entails selecting the missing piece to complete a matrix or pattern. Contributes to the Fluid Reasoning Index (FRI).
WISC-V	Visual puzzles	Requires choosing pieces to finish a displayed puzzle under timed conditions. Contributes to the VSI.
WISC-V	Digit Span	Involves recalling number sequences in forward, backward, and ascending order. Contributes to the Working Memory Index (WMI).
WISC-V	Picture Span	Consists of recognizing previously viewed images in a specified sequence. Contributes to the WMI.
WISC-V	Figure weights	Solves an arithmetic problem using visual hints under timed constraints. Contributes to the FRI.
WISC-V	Coding	Involves copying symbols matched to simple shapes or numbers. Contributes to the Processing Speed Index (PSI).
WISC-V	Symbol search	Requires quickly identifying and marking symbol sequences. Contributes to the PSI.
WISC-V	Comprehension	Evaluates understanding of general principles in social scenarios. Supplementary subtest.
NEPSY-II	Emotion recognition	Assesses the ability to discern and match facial expressions representing six basic emotions. Non-verbal task focusing on emotional perception.

As shown in Table 1, the WISC-V provides a multi-dimensional measure of intellectual functioning through 10 core subtests, each tapping into different domains such as verbal comprehension, visuospatial skills, fluid reasoning, working memory, and processing speed. The NEPSY-II, meanwhile, specifically targets socio-cognitive processes with subtests that measure emotion recognition and theory of mind.

#### 2.3.1 Wechsler intelligence scale for children—fifth edition (WISC-V)

The intellectual capabilities of children were comprehensively evaluated using the WISC-V, which comprises 10 core subtests. These subtests include Similarities (identifying the commonalities between two objects or concepts), Vocabulary (naming and defining words using visual and auditory cues), Block Design (assembling bi-colored blocks to replicate a given model within a time limit), Matrix Reasoning (selecting a piece to complete a matrix or pattern), Visual Puzzles (choosing pieces to finish a displayed puzzle within a time limit), Digit Span (recalling number sequences in forward, backward, and ascending order), Picture Span (recognizing previously seen images in sequence), Figure Weights (solving an arithmetic problem using visual hints within a time limit), Coding (copying symbols matched to simple shapes or numbers), and Symbol Search (quickly identifying and marking a symbol sequence).

These subtests contribute to five composite scores, each representing a distinct aspect of intellectual abilities: Verbal Comprehension Index (VCI), Visual Spatial Index (VSI), Fluid Reasoning Index (FRI), Working Memory Index (WMI), and Processing Speed Index (PSI). The VCI, derived from Similarities and Vocabulary subtests, measures verbal understanding and conceptual reasoning. The VSI, based on Block Design and Visual Puzzles subtests, assesses spatial and visual perception. The FRI, obtained from Matrix Reasoning and Figure Weights subtests, represents the ability to solve novel problems and think logically. The WMI, derived from Digit Span and Picture Span subtests, indicates the capacity to mentally manipulate and retain information. The PSI, based on Coding and Symbol Search subtests, measures the ability to quickly and accurately process simple, routine tasks. Additionally, the supplementary test “Comprehension” was conducted to measure the understanding of general principles in social situations among children. The Full Scale Intelligence Quotient (FSIQ) is calculated from the combined performance of these indices, providing an estimate of the participant’s overall intellectual capability.

We chose to work using the Wechsler scales given that they are backed by extensive normative data, standardized across diverse populations, ensuring that the results are accurate and comparable across different demographic groups (Benedict et al., 2012). This is particularly important when assessing individuals with unknown cognitive disorders, as it allows clinicians to interpret scores within the context of a well-defined normative framework. For example, studies have shown that the WAIS is effective in identifying cognitive deficits in populations with various conditions, including schizophrenia and traumatic brain injury, by providing a clear profile of cognitive functioning (Allen et al., 2010; Johansen et al., 2011).

#### 2.3.2 Neuropsychological assessment—second edition (NEPSY-II)

The NEPSY-II (Korkman et al., 2012) was used to evaluate socio-cognitive functions in participants through two subtests. The evaluation of social perception includes subtests Emotion Recognition (RE) and Theory of Mind (TE). Emotion Recognition assesses the ability to differentiate between common facial expressions, while Theory of Mind evaluates an individual’s capacity to understand others’ perspectives both on verbal and visual modalities.

The Emotion Recognition subtest evaluates a child’s ability to discern and match facial expressions associated with six basic emotions: happiness, sadness, fear, anger, disgust, and neutrality. Participants are required to compare facial expressions and determine whether they are identical or different, as well as identify pairs of faces that exhibit the same emotion. This subtest is entirely non-verbal, designed to minimize the influence of linguistic proficiency and instead focus on the child’s capacity for visual discrimination and emotional perception. Performance in this task reflects the child’s ability to interpret emotional cues, which is a foundational skill for effective social interactions. Deficits in this area can manifest as difficulty recognizing emotional states in others, contributing to misunderstandings, social withdrawal, or inappropriate responses in social contexts.

In contrast, the Theory of Mind subtest measures a child’s ability to understand the mental states, beliefs, and intentions of others, as well as how these mental states influence actions and behaviors. The task includes two complementary components. In the verbal reasoning tasks, participants are presented with scenarios, either narrated or depicted visually, and asked to infer the thoughts or emotions of the characters involved. The contextual reasoning tasks use visual stimuli to assess the ability to link contextual social cues with corresponding emotional states or intentions. Together, these components evaluate the child’s capacity to infer social meaning, predict behaviors based on others’ perspectives, and understand that others may hold beliefs or emotions different from their own. This skill is critical for successful interaction within social environments, as it allows individuals to adapt to the dynamic demands of social exchanges.

We chose to work using the NEPSY-II given that its proficiency to discriminate between different cognitive disabilities, making it a valuable tool for identifying cognitive deficits in children with varying backgrounds (Glass et al., 2015; Niutanen et al., 2022). The assessment’s normative data, stratified by age, sex, and other demographic factors, further enhance its applicability in clinical settings, allowing for accurate interpretations of individual scores (Glass et al., 2015; Irvine et al., 2023). Moreover, the NEPSY-II has been shown to be effective in various clinical populations, including children with Down syndrome, autism spectrum disorder, and those with prenatal exposure to substances like alcohol (Schworer et al., 2021; Soltani et al., 2023). Its flexibility allows clinicians to tailor the assessment to the specific needs of the child, which is capital when dealing with unknown cognitive pathologies. This adaptability is further evidenced by its application in research settings, where it has been used to explore cognitive development in relation to environmental factors, such as maternal nutrition and prenatal exposure to toxins (Cruz-Rodríguez et al., 2024; Irvine et al., 2023).

### 2.4 TEST-ASAP

Inspired by the work of Gerardin-Collet (1999) within the framework of Interlocutory Analysis of Autism-Spectrum Disorder children, the Trognon Ecological Side Task for the Assessment of Speech-Act Processing (TEST-ASAP) is designed to capture social dynamics that naturally emerge during test sessions. Rather than functioning as a separate instrument, the TEST-ASAP is embedded into routine evaluation tasks so that speech acts, inferred requests, and cooperative behaviors can be observed in a more authentic, ecologically valid context. Building on these principles, the TEST-ASAP integrates insights from the 2TK model to systematically analyze how individuals weigh social costs and benefits in a real context.

#### 2.4.1 The “Basket” subtask: cooperation under instruction, high-cost interaction, low benefits

The “Basket” task in the TEST-ASAP involves providing the participant with a shortened and blunt pencil during tests requiring fine motor skills, like the Drawing Fluency in NEPSY-II or Barrage in WISC-V. The experimenter presents the pencil, remarking, “Here, for this exercise, you need a pencil,” and then adds, “It’s badly sharpened. Here’s a sharpener, but you’ll need to fetch the garbage can to use it.” This setup makes the necessary actions clear, reducing the need for inference. Since the pencil is still functional, the value of sharpening it is low, suggesting that compliance would be mainly to reciprocate the experimenter’s efforts to ensure optimal testing conditions.

Should there be no cooperative action, the experimenter makes a secondary prompt: “You should sharpen your pencil; you work better with a well-sharpened pencil,” which again suggests but does not enforce compliance. If cooperation still fails to manifest, the experimenter then creates a scenario requiring direct assistance by pretending to need the pencil themselves and asking, “Can you get me the wastebasket so I can sharpen my pencil?” This progression of requests serves to measure the degree of cooperation or its absence in a graded manner.

#### 2.4.2 The “Door” subtask: inference of inter-subjective agentivity, low-cost interaction, low benefits

The “Door” task of the TEST-ASAP, the experimenter distributes materials to parents outside and intentionally leaves the door open upon returning. From a distant desk, the experimenter exclaims, “Oh, I forgot to close the door!” This statement requires the participant to infer that the remark, although seemingly directed to no one in particular, is a request for them to close the door. Thus, we thought that the participant is likely to comply given the low effort involved and the greater inconvenience to the speaker.

If unheeded, a clearer prompt follows: “We really should close that door.” This reduces the need for inference by specifying the action but still requires the participant to recognize that “we” includes him. This step examines inter-subjective agency, a concept important in understanding social interactions impaired in autism spectrum disorders (Gerardin-Collet, 1999).

Should these indirect cues fail, the experimenter directly asks, “Could you go and close the door, please?” This explicit instruction minimizes the need for inference, relying primarily on the participant’s engagement and willingness to cooperate.

#### 2.4.3 The “Syllogism” subtask: logical inference, low-cost interaction, high benefits at the cost of infringing social norms

The “Syllogism” task assesses basic logical inference within the context of social norms, posing an interesting challenge despite its simple deductive requirements. Scheduled just before the lunch break between two psychometric sessions, the task begins with the experimenter’s announcement: “Well, this is our last test, then we’ll be done.” It then links the conclusion of this test with the participant’s next action: “When we are done, you can go straight to your parent to get lunch.” This sequence sets up a straightforward logical progression—ending the test leads directly to the session’s end and the subsequent action of seeking one’s parent.

After the psychometric test’s completion, the experimenter states “That was the last test,” and monitors the participant for any initiative to leave. Despite the simplicity of the inferences required, the task probes the participant’s willingness to break a typical social convention: not leaving the (asymmetrical) interaction without explicit dismissal. If the participant does not respond, a clearer reminder is issued: “That was the last test, we are finished.” Should inactivity persist, a final, unambiguous command is given: “Since we have finished, can you go and get your parent now?” This directive explicitly states the expected action, eliminating the need for inference, emphasizing the anticipated cooperative behavior, with coherence to social norms. Thus, this specific task aligns logical reasoning with pragmatic social strategies, highlighting how individuals economically leverage their interactions for personal benefit.

### 2.5 Computational methods used in the study: the recurrent neural network

To model and predict the dynamics of dialogical interactions in Shwachman-Diamond Syndrome (SDS) patients, we employed a Recurrent Neural Network (RNN). RNNs are a class of machine learning models specifically designed to handle sequential data, making them particularly suitable for analyzing time-structured datasets such as conversational exchanges (Dutta Baruah and Muñoz Organero, 2024; Mienye et al., 2024). Their key feature is the ability to maintain a memory of past inputs through a recurrent connection within their hidden state, allowing the network to capture dependencies between successive elements of a sequence (Tsantekidis et al., 2022).

In this study, the RNN processed interaction data, with epsilon (*ε*), representing the chronological order of speech acts, used as the input, and vergence (*V*), representing the degree of cooperation or alignment between interlocutors, as the predicted output. The hidden state of the RNN was iteratively updated to incorporate the influence of previous ε values, thereby modeling the progression of interaction dynamics over time.

The computational process of the RNN model used can be summarized as follows:

At each time step **ε**, the network updates its internal hidden state *h_ε_* based on the current input (dialogical context) and the previous hidden state *h_ε-1_*. The update is thus computed using the following formula:


$$h_{\epsilon }=\sigma_{h}\left(W_{xh}\epsilon +W_{hh}h_{\epsilon -1}+b_{h}\right)$$

where:

- *W_xh_* is the weight matrix connecting the input (speech act index *ε*) to the hidden state.
- *W_hh_* represents the recurrent connections within the hidden layer.
- *b_h_* is the bias term.
- *σ_h_* is the activation function, which bounds the outputs and captures non-linear relationships.

Thus, the predicted vergence (*V_ε_*) at time step *ε* is computed as:


$$V\varepsilon =\sigma_{y}\left(W_{hy}h_{\varepsilon }+b_{y}\right)$$

where:

- *W_hy_* maps the hidden state to the output layer.
- *B_y_* is the bias for the output.
- *σ_y_* is the activation function applied at the output layer.

In this experiment, the RNN was trained on interaction data from experimental tasks. Each sequence consisted of a series of dialogical turns indexed by *ε*, paired with corresponding vergence values, which represent the alignment between interlocutors at that point in the interaction. Training the network allowed it to model and predict how dialogical alignment should evolves over time, given the dynamic that was observed previously.

## 3 Model

### 3.1 Elements of 2TK model

The topological and kinetic model (2TK), developed by Trognon (2022), offers a unified analytical framework for examining dialogic interactions. The model synthesizes principles from three major traditions in discourse analysis: Ludics, which provides structural representations of dialogue elements (Girard, 1987, 2001); Segmented Discourse Representation Theory (SDRT), which models the temporal dynamics and transitions of discourse (Asher and Lascarides, 2003); and Interlocutory Analysis, which focuses on the functional units of speech acts within their pragmatic context (Searle, 1969; Grice, 1975). By integrating these approaches, the 2TK model overcomes the limitations of individual methodologies, offering a robust and versatile tool for researchers and clinicians alike.

The foundation of the 2TK model is its capacity to represent dialogues as dynamic systems composed of hierarchical structures. These structures include macro-level plans, intermediate-level concatenations (sequences of related discourse units), and individual speech acts, each characterized by specific parameters such as type, polarity, and temporal position. This multi-layered architecture allows the 2TK model to capture the complexity of interactional dynamics, including the interplay of cooperative and divergent behaviors.

A key metric in the 2TK framework is vergence, which measures the degree of convergence between interlocutors. Vergence is calculated as the ratio of positively polarized speech acts—those that contribute toward a shared dialogic goal—to the total number of speech acts within a given segment. Values above 0.5 indicate effective cooperation, while lower values suggest divergence. Complementing this measure, celerity evaluates the efficiency of the dialogue by quantifying the proportion of necessary and decisive speech acts advancing the dialogical goal relatively to the total produced. The relationship between vergence and celerity is formalized in the Primacy of Celerity Theorem, which posits that vergence cannot exceed celerity, highlighting efficiency as an upper boundary for conversational convergence.

The 2TK model operates through a modular and parametric function, the Interaction Function, denoted by Ψ(*ξ*, *ε*, *σ_n_*), which serves as the foundational formula of the 2TK model. It represents the state of the dialogical system as a function of dialogical time (i.e., the statement, the speech act, or the indexical, depending on the resolution).

Expressed as Ψ(*ξ*, *ε*, *σ_n_*), *ξ* denotes the topological position of speech acts, ε represents the iterative dialogic time, and *σ_n_* encapsulates the parameters defining the state of the system, including rhetorical relations and the type of acts performed. This formulation provides a flexible foundation for analyzing dialogic processes across diverse contexts.

One of the model’s distinguishing features is its dual applicability. On the one hand, it supports computational analysis, enabling the automated examination of complex interactions through algorithmic tools. On the other hand, it remains accessible for manual implementation, allowing clinicians and researchers in resource-limited environments to leverage its insights without specialized software or hardware. This adaptability ensures that the 2TK model can bridge the gap between theoretical research and practical application, offering valuable insights into the dynamics of interaction in both experimental and clinical settings.

By harmonizing structural, temporal, and pragmatic dimensions of dialogue, the 2TK model could advance the study of discourse beyond the capabilities of existing methodologies. Its ability to integrate and quantify multiple aspects of interactional dynamics makes it a powerful instrument for understanding cooperative and divergent behaviors in naturalistic and clinical contexts.

### 3.2 The hierarchical structure of dialogue in the 2TK model

In the 2TK framework, a dialogue is represented as a sequence of planes (*Π*), the largest units of analysis beside the whole dialogue, encompassing sequences of related conversational themes. Planes themselves are composed of smaller units called concatenations ℭ representing intermediate structures grouping speech acts that collectively contribute to a specific subgoal or task within the plane. These concatenations group sequences of related speech acts (*κ*), which are the fundamental building blocks of dialogue, which is defined by the content it conveys (e.g., information) and its type (see below).

Thus, speech acts are classified into five main types, each reflecting distinct communicative functions:

- DO: An action performed, verbally or non-verbally (e.g., giving an object).
- MK (make-know): Informational statements (e.g., describing an object).
- DMK (do make-know): Requests for information (e.g., asking a question).
- DC (do can): Invitations or offers, where an interlocutor has a choice (e.g., “Would you like...?”).
- DM (do must): Directives that leave no alternative (e.g., “You must do this”).

We adapted our speech act classification from (Caelen, 2003) taxonomy, which itself builds on (Searle and Vanderveken, 1985) work. In his approach—initially devised for man–machine dialogue—each act is classified according to its illocutionary force (e.g., “FA” for performing an action, “FF” for requesting an action, “FS” for communicating information). This simplified scheme retains Searle & Vanderveken’s foundational distinctions (directive, assertive, etc.) but labels them with functional codes, thus making it particularly amenable to computational dialog analysis.

### 3.3 Kinetic and topology

Every speech act within a dialogue is situated in a topological and temporal space. The address (*ξ*_(*x,y*)_) represents the spatial coordinates of the act within the dialogue’s structure, while the dialogical time *ε* tracks the order of each act relative to the ongoing exchange or dialogical structure.

### 3.4 Relevant properties of speech acts and associated metrics

In the 2TK framework, each speech act *κ* is characterized by two fundamental properties among others: polarity and decisiveness, which together provide a detailed account of its role within the dialogue. These properties allow the model to evaluate how each act contributes to the overarching interaction, both in terms of alignment with dialogue goals and the resolution of specific tasks.

The Polarity (*δ*) represents the provability of a speech act within the dialogic structure, as determined by its alignment with a given object of analysis. This concept, derived from Ludics, evaluates whether the content of a speech act substantiates or supports the discourse relative to a specific object. In essence, polarity assesses whether a speech act is able to “prove” that the current dialogue refers to a particular topic, task, or interactional context.

For example, consider a dialogue where the object of analysis is a discussion about a cat. If one interlocutor states, “My cat is orange,” this act has positive polarity (*δ* = 1) because it directly supports the provability of the dialogue’s alignment with the topic of a cat. Furthermore, it substantiates potential questions such as “What color is your cat?” or “Do you have a cat?” Conversely, if the interlocutor states, “I have a green bicycle,” this act cannot be used to prove that the dialogue is centered around the object “cat” and thus has null polarity (*δ* = 0).

Polarity thus operates as a broad and flexible concept, applicable across various objects of analysis, as the object might be task-specific (e.g., the polarity of a speech act may indicate whether it aligns with the resolution of a task, like asking for a calculator during a math problem-solving task); or even interpersonal (e.g., reflect alignment with an interlocutor’s contributions, such as responding directly to a question or request).

In practical terms, a positively polarized speech act proves its relevance to the object, maintaining or advancing the coherence of the dialogue. A negatively polarized act, by contrast, represents a divergence, failing to contribute to the dialogic system’s alignment with the chosen object. This characterization of polarity enables the 2TK framework to systematically evaluate the coherence and focus of interactions in a nuanced and analytically rigorous manner.

In contrast, the Decisivity (*d*) evaluates the direct contribution of a speech act to task resolution. A speech act is deemed decisive (*d* = 1) if it directly advances the completion of the current task or goal, such as providing a correct answer or performing an essential step in the interaction. Decisive acts are critical for achieving dialogue objectives efficiently. In contrast, non-decisive acts (*d* = 0) play a more peripheral role; they may offer Supplementary information, introduce context, or even cause digressions without directly resolving the task at hand.

By combining these two properties, the 2TK framework captures the nuanced ways in which speech acts interact with their objects of analysis, whether these are task-oriented or interpersonal. This dual-layered characterization enables a precise analysis of alignment, divergence, and efficiency in dialogic interactions, providing insights into both cooperative dynamics and the potential sources of miscommunication.

Those two properties allows to compute two key metrics, namely Vergence and Celerity, that we will use later in the present work.

The Vergence (*V*) quantifies the degree of cooperation and alignment between interlocutors in a dialogue. This metric reflects how speech acts contribute to the shared objectives of the interaction, particularly in terms of maintaining coherence and focus. Vergence is calculated as the proportion of positively polarized speech acts relative to the total number of speech acts in a dialogue or segment:


$$V=\frac{\sum \kappa_{i,\delta \left(1\right)}}{\sum \kappa_{i}}$$

Here, $\kappa_{i,\delta \left(1\right)}$ denotes speech acts that are positively polarized, meaning they substantiate the relevance of the dialogue relative to the given object of analysis, as determined by their polarity ( δ=1 ). The denominator, $\sum \kappa_{i}$ , represents the total number of speech acts in the analyzed segment. A vergence value “*V*” greater than 0.5 indicates cooperation, with most acts contributing positively to the dialogue’s shared objectives, while a value of *V* < 0.5 suggests divergence or misalignment. Maximal vergence (*V* = 1) occurs when all speech acts in the segment are positively polarized, demonstrating full alignment and cooperation.

Similarly, the Celerity (*C*) assesses the efficiency of a dialogue by evaluating how directly the speech acts contribute to task resolution. It measures the proportion of decisive speech acts relative to the total number of speech acts, where decisive acts (*d* = 1) directly advance the completion of the task or goal at hand. Celerity is defined as:


$$C=\frac{\sum \kappa_{i,d\left(1\right)}}{\sum \kappa_{i}}$$

In this formula, $\kappa_{i,d\left(1\right)}$ denotes speech acts classified as decisive, and $\sum \kappa_{i}$ is the total number of speech acts in the segment. High celerity indicates an efficient dialogue where the majority of speech acts are task-oriented and purposeful. Conversely, lower celerity reflects inefficiency, often due to digressions, redundant contributions, or irrelevant exchanges.

The relationship between these two metrics is governed by the Primacy of Vergence Theorem, which states:


C≤V

This theorem underscores that interlocutors’ cooperation (vergence) is a prerequisite for efficiency (celerity) within the dialogue. A cooperative and aligned interaction is essential to achieve high efficiency, as misaligned exchanges reduce the likelihood of task-focused speech acts. In essence, dialogue efficiency is inherently limited by the degree of cooperation present.

To apply these metrics in practice, each speech act is annotated with its polarity and decisiveness based on its contribution to the dialogue. These annotations are aggregated across the selected segment of analysis—whether it be a concatenation, plan, or the dialogue as a whole—and the formulas for *V* and *C* are applied. For example, in a task-oriented interaction where interlocutors remain focused on shared objectives, both *V* and *C* would approach 1, indicating high levels of cooperation and efficiency. In contrast, a dialogue characterized by frequent digressions or conflicting objectives would exhibit lower values for both metrics, signaling divergence and inefficiency.

More specifically, given that vergence allows for the measurement of interlocutors’ cooperation in task resolution or object orientation, and celerity allows for the measurement of efficiency in task resolution or discursive progression, the comparison of these two measures highlights the impact of inter-individual cooperation on task resolution. Indeed, if the interlocutors do not produce speech acts directed toward the same object (i.e., low vergence), then its resolution will necessarily be hindered (i.e., low celerity). In contrast, two interlocutors may actively attempt to resolve the current task (i.e., high vergence), but may encounter mutual comprehension difficulties or with the task itself, for example, through the production of repetitions (i.e., low celerity). Moreover, these two measures being carried out on the same time scale, they are accessible to statistical measurement by Pearson correlation.

### 3.5 An example of encoding in the 2TK model

This section demonstrates how the 2TK model encodes thematic organization within a dialogue, using an excerpt from The Count of Monte Cristo by Dumas (1849). The selected passage features an exchange between Abbé Faria and Edmond Dantès, wherein Faria questions Dantès about the life events leading to his unjust imprisonment. This corpus is a canonical example frequently analyzed within Ludics (Fouqueré et al., 2018 Lecomte and Quatrini, 2011), making it an ideal candidate for illustrating the topological and kinetic analysis enabled by the 2TK model.

#### 3.5.1 Corpus

Table 2 presents the adapted text from Dumas (1849) as used in prior analyses (Fouqueré et al., 2018 Lecomte and Quatrini, 2011). The dialogue consists of eight speech acts that organize into two thematic units (i.e., two concatenations). The first theme addresses Edmond’s ascension to the captaincy of the Pharaoh, while the second focuses on his impending marriage. Together, these concatenations form a single dialogic plan.

**Table 2:** Corpus adapted from (Fouqueré et al., 2018 Lecomte and Quatrini, 2011). Written initially by Dumas (1849).

Locutor	Enunciation	*ε*
Faria	What was your life at that time?	1
Edmond	I was going to become captain of the Pharaoh.	2
Edmond	I was going to marry a beautiful girl.	3
Faria	Was there anyone who had a vested interest in you not becoming captain of the Pharaoh?	4
Edmond	[...] only one man [...]	5
Faria	What was his name?	6
Edmond	Danglars	7
Faria	So... now tell me about this beautiful young lady...	8

#### 3.5.2 Topological encoding

The topological structure of the dialogue is encoded by mapping speech acts onto a hierarchical representation. Themes (or concatenations) are incremented along the x-axis, representing the emergence or elaboration of new topics (e.g., “Becoming captain of the Pharaoh” or “Getting married to a beautiful woman”). In contrast, increments along the y-axis indicate contributions that deepen the discussion of a specific theme (e.g., “What was his name?” → “Danglars”). It can thus be represented in a topological matrix congruent with this described structure (Table 3).

**Table 3:** Topological encoding of the exchange.

x/y	*x* = 1	*x* = 2
*y* = 1	What was your life at that time?	
*y* = 2	I was going to become captain of the Pharaoh.	I was going to marry a beautiful girl.
*y* = 3	Was there anyone who had a vested interest in you not becoming captain of the Pharaoh?	So... now tell me about this beautiful young lady...
*y* = 4	[...] only one man [...]	
*y* = 5	What was his name?	
*y* = 6	Danglars	

#### 3.5.3 Polarity and vergence

To compute polarity, we first define the object of provability. Here, we evaluate each speech act’s relevance to resolving the ongoing task, using the question: “Does this speech act inferentially contribute to the resolution of the current task?” For example, at *ε* = 7, the act “Danglars” directly answers prior inquiries at *ε* = 6 (“What was his name?”) and *ε* = 4 (“Was there anyone who had a vested interest in you not becoming captain of the *Pharaoh*?”). Thus, this act positively proves its relevance to the task.

Applying this rule, we find that all speech acts in the excerpt exhibit positive task polarity (*δ* = 1). The vergence *V* is then calculated as the ratio of positively polarized acts to the total number of acts:


$$V=\frac{\sum \kappa_{i,\delta =1}}{\sum \kappa_{i}}=\frac{8}{8}=1$$

Here, *V* = 1, indicating a maximal convergence. This reflects maximal cooperation between interlocutors in advancing the task.

#### 3.5.4 Decisivity and celerity

While all speech acts contribute to the dialogue, not all are decisive in resolving the task. For instance, at *ε* = 5, Edmond responds, “Only one man,” to Faria’s question at *ε* = 4 (“Was there anyone who had a vested interest...?”). This response is redundant, as it reiterates the need for identification already implied in the question. Similarly, *ε* = 6 (“What was his name?”) would have been unnecessary if the name “Danglars” (*ε* = 7) had been provided immediately. These acts are thus coded as non-decisive (*d* = 0), while all others are coded as decisive (*d* = 1).

Celerity “*C*,” the efficiency metric, is then calculated as the ratio of decisive speech acts to the total number of acts:


$$C=\frac{\sum \kappa_{i,d=1}}{\sum \kappa_{i}}=\frac{6}{8}=0.75$$

In this example, *C*, = 0.75, indicating that the dialogue have progressed 25% slower when compared to the situation where all non-decisive acts would be omitted.

Thus, the combination of vergence and celerity highlights the cooperative yet somewhat inefficient nature of this exchange. While the interlocutors demonstrate full alignment in achieving their shared goal (*V* = 1), the celerity score reveals opportunities for streamlining the interaction. This analysis illustrates the 2TK model’s capacity to disentangle cooperative dynamics from task efficiency, offering nuanced insights into dialogic processes.

## 4 Results

### 4.1 Statistics

Table 4 provides a detailed overview of the statistics for the study participants, comparing two individual cases (CG and MC) with group averages for the Shwachman-Diamond Syndrome (SDS) cohort (*n* = 5) and matched controls (*n* = 5). The variables include age, gender distribution, and various cognitive and socio-emotional measures. The *p*-values indicate statistical significance for comparisons between the SDS and control groups. It was observed that patients with Shwachman-Diamond syndrome recorded lower scores on all variables, when compared to matched controls.

**Table 4:** Descriptive statistics of the study sample (VC, verbal comprehension; WM, working memory; FR, fluid reasoning; VS, visuo-spatial; PS, processing speed; GAI, general ability index; CPI, cognitive proficiency index; IQ, intellectual quotient; E.R., emotion recognition; ToM-V/C, theory of mind – verbal/contextual; PR, percentile rank).

Parameter	CG	MC	SDS (*n* = 5)	Controls (*n* = 5)	*p*-value
Age	8	11	11.35(±1.87)	10.82(±2.16)	0.69
Male	–	X	3	3	–
Female	X	–	2	2	–
VC	68	103	85.6(±17.9)	109.8(±8.49)	***0.002***
WM	110	115	96.6(±20)	110.6(±13.27)	0.22
FR	106	97	87.2(±19.56)	103(±11.02)	0.15
VS	97	86	83.8(±15.59)	114.2(±4.88)	***0.003***
PS	132	111	100.8(±23.62)	101.2(±9.83)	0.97
GAI	87	95	85.53(±16.51)	109(±9.21)	***<0.001***
CPI	124	116	98.7(±20.75)	105.9(±12.08)	0.72
IQ	94	96	83.4(±18.83)	109.8(±12.02)	***<0.001***
E.R.	11	12	8.75(±2.86)	10(±3.57)	0.25
ToM-V	PR: 2–5	PR: 11–25	5.4(±6.39)	64(±30.07)	***<0.001***
ToM-C	PR: 6–10	PR: 11–25	5.8(±6.67)	55(±31.56)	***<0.001***

Bold *p*-values indicate statistically significant results (*p* < 0.05).

The mean age for the SDS group (11.35 ± 1.87 years) and controls (10.82 ± 2.16 years) shows no significant difference on the *t*-test (*p* = 0.69). Gender distribution is balanced across groups, with three males and two females in both cohorts. The ANOVA test on cognitive measures reveals significant deficits in the SDS group compared to controls, particularly in Verbal Comprehension (VC: 85.6 ± 17.9 vs. 109.8 ± 8.49, *p* = 0.002), Visuo-Spatial Index (VS: 83.8 ± 15.59 vs. 114.2 ± 4.88, *p* = 0.003), General Ability Index (GAI: 85.53 ± 16.51 vs. 109 ± 9.21, *p* < 0.001), and overall Intellectual Quotient (IQ: 83.4 ± 18.83 vs. 109.8 ± 12.02, *p* < 0.001). Working Memory (WM: *p* = 0.22), In contrast, Fluid Reasoning (FR: *p* = 0.15), and Processing Speed (PS: *p* = 0.97) show no statistically significant differences between groups, although mean scores for SDS participants tend to be lower.

Socio-emotional measures, including Emotion Recognition (E.R.) and Theory of Mind (ToM), highlight marked impairments in the SDS group. While Emotion Recognition scores (E.R.: 8.75 ± 2.86 vs. 10 ± 3.57, *p* = 0.25) did not differ significantly, Theory of Mind–Verbal (ToM-V: percentile rank 5.4 ± 6.39 vs. 64 ± 30.07, *p* < 0.001) and Theory of Mind–Contextual (ToM-C: percentile rank 5.8 ± 6.67 vs. 55 ± 31.56, *p* < 0.001) demonstrate substantial deficits in SDS children.

These data collectively underscore the multidimensional nature of cognitive and socio-emotional impairments in Shwachman-Diamond Syndrome, with pronounced challenges in verbal comprehension, visuo-spatial processing, general intellectual ability, and advanced theory of mind tasks.

### 4.2 Case studies

#### 4.2.1 Pragmatics and dialogic strategy: toward an economic perspective in a case study

The case of CG provides an insightful example of the challenges parents face in certain interactive situations, such as homework completion at home. Homework often sparks intrafamily conflicts within the SDS context, with parents frequently citing uncooperative behavior. Prior to undertaking the initial nine items of the WISC-V “Comprehension” subtest, CG expressed her frustration for the last exercise postponing her lunch break (Table 5).

**Table 5:** Exchange that preceded the initiation of the “Comprehension” subtest of the WISC-V.

Locutor	ε	Enunciation
CG	–5	Hey hey hey so we have one more and then we are almost done
CG	–4	Oh no!
Exp	–3	Yeah that’s good
Exp	−2	In any case with the tablet we will have finished...
CG	−1	No! And I do not have one left, I have several!

#### 4.2.2 Convergence of interlocutors

**Figure 1:**
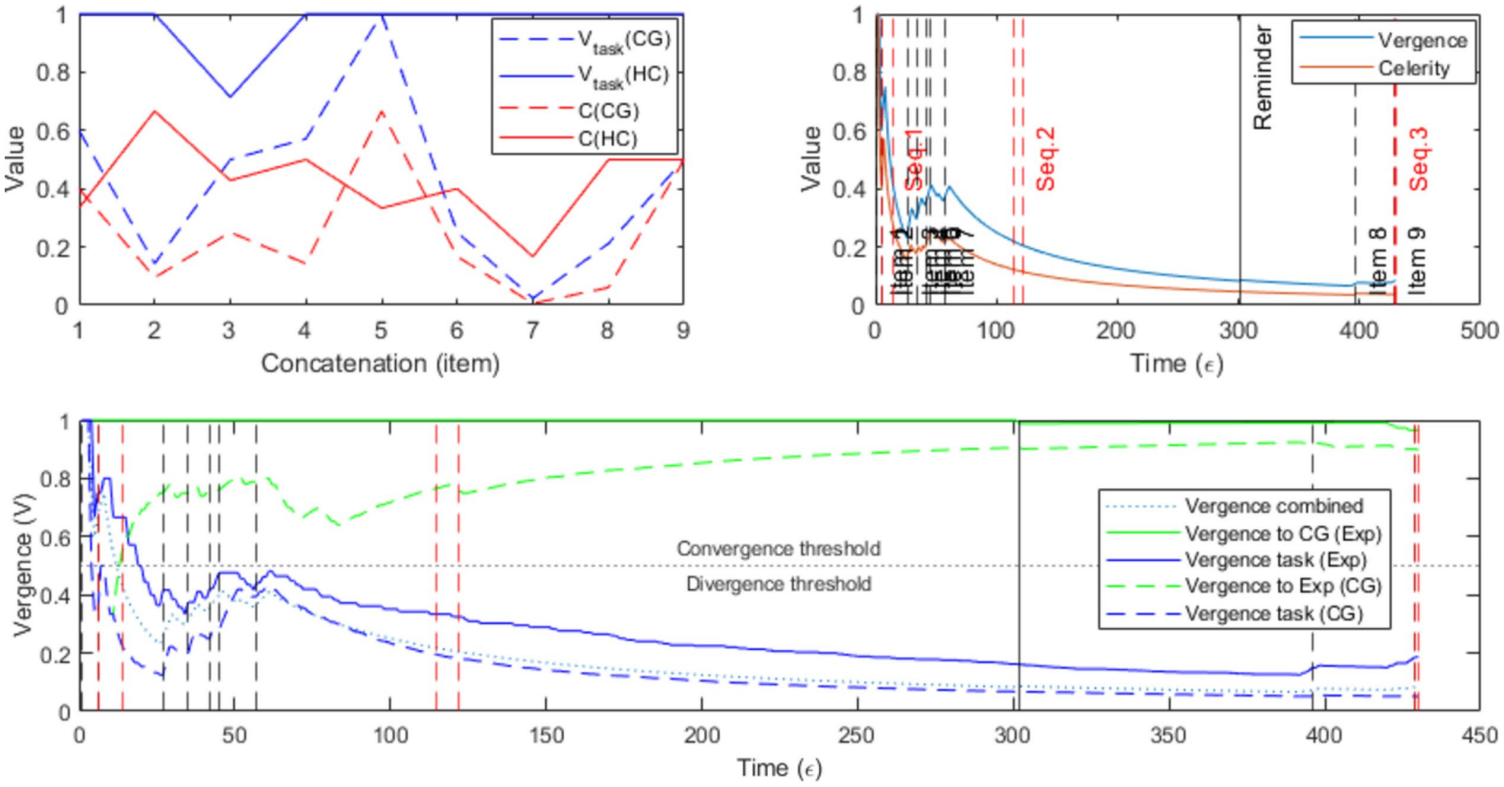
Metrics-Based Analysis of Experimenter-CG interaction during the ‘Comprehension’ subtest of the WISC-V in 2TK model. The upper left panel shows vergence (V) and celerity (C) values for each item (encoded as a concatenation) for both the patient (dashed line) and the matched control (solid line). The upper right panel details vergence (blue) and celerity (orange) measurements across the interaction for the subject CG and highlights segmented sequences. The lower panel depicts vergence based on two parameters: task-oriented vergence (i.e., when the linguistic act is directed toward solving the task, shown in blue; *V_Task_*) and interlocutor-oriented vergence (i.e., when the linguistic act aims to validate the last ratified concatenation by the interlocutor). Seq. corresponds to a given sequence.

Figure 1 presents the analysis of dialogue dynamics associated with the ‘Comprehension’ subtest completion, emphasizing vergence. Data illustrated the impact of the self-benefits on the dialogical system in the context of SDS.

Administering the “Comprehension” subtest proved particularly challenging due to the patient’s numerous spontaneous comments, which were pragmatically related to the thematic content of the items to varying degrees. From the onset of the first item, a significant amount of spontaneous commentary followed the confirmation of the subject’s response. This led to a decrease in task-related vergence at the first concatenation (i.e., all speech act for a given item), dropping to 0.6 (i.e., 40% of speech act were not directed toward solving the task), and subsequently to 0.15 for the second concatenation, indicating a significant divergence of the dialogical system from the ongoing task (see Figure 1, left; Figure 1, right, Seq.1; Table 6).

**Table 6:** Corpus associated with Seq.1 in Figure 1.

Locutor	ε	Enunciation
Exp	6	Why cannot you pet a dog you do not know?
CG	7	Otherwise it bites you if it is mean
Exp	8	Yeah that’s good
CG	9	Because otherwise it bites your cheek
CG	10	That’s what dad had when he was young when he had seen a dog that was very bad
Exp	11	Yeah
CG	12	And he wanted to pet it
CG	13	And then he had his cheek bitten
CG	14	And so we sewed him up

Subsequently, the experimenter attempted to promptly close any tangential sub-themes initiated by the patient. This approach enabled the vergence to return to an optimal level of convergence at the 5th concatenation (*V* = 1, Figure 1, Left). However, this level could not be sustained during the following item, where the vergence significantly decreased again (*V* = 0.25, Figure 1, Left). Additionally, at the next item, the patient connected a personal anecdote to the thematic field of the question (i.e., emergency numbers), progressively monopolizing the dialogic system to a point where she completely ignored her interlocutor’s interventions (Figure 1, Seq.2, Right; Table 7).

**Table 7:** Corpus associated with Seq.2 in Figure 1.

Locutor	ε	Enunciation
CG	115	then we have Marie Helene she called late
CG	116	and then twice the EMS
CG	117	EMS and the second time they say
CG	118	“but what is actually going on”
CG	119	we said “no no it’s fixed”
Exp	120	hey, hey, hey, come on, we have got 5 min left, let us do the ones that are left?
CG	121	so it’s normal that Claire goes no more on the table
CG	122	Marie-Hélène does it but only on the ladder

Later, the patient was so absorbed in her additional themes that she neglected the task entirely (Figure 1, Right, Seq. 3), breaking the communication link and leading the experimenter to halt the task to start the lunch break (Table 8).

**Table 8:** Corpus associated with Seq.3 in Figure 1.

Locutor	ε	Enunciation
Exp	429	so why is it good for kids to help with chores [trad: tâches ménagères] around the house?
CG	430	uh what about the shelf [trad: étagère]?

Comparison with a matched control subject is even more stringent when considering the information provided by celerity. Celerity solely measures the performative aspect of the task by recording only the decisive question-answer pairs, excluding any superficial continuations and elaborations. In contrast, vergence assesses the directive nature of the speech act toward a task resolution element within the dialogic system, thereby retaining additional elaborations provided they were made before the correct response was ratified. It is observed that in the case of CG, reductions in celerity almost always coincide with a decrease in vergence due to two types of occurrences: additional elaborations post-response ratification, ending the current concatenation (i.e., the item; Seq.1), or divergences that are only semantically related to the ongoing task (Seq.2).

In the case of the control subject (HC), task vergence typically remains optimal (*V* = 1; Figure 1, Left), except during the third concatenation where a divergence occurred: the experimenter prematurely ratified the subject’s response, interrupting their subsequent speech act due to theme closure, thus creating initial divergence between the speakers. This was followed by another ratification of theme closure due to the subject’s incomplete statement, leading to a second divergence (Seq.HC1). Notably, the sequence was already marked by reduced celerity at this concatenation (Figure 1, Left, Concatenation 3; Table 9), caused by environmental noise disrupting the question’s articulation, which likely influenced the rest of the sequence (Table 9).

**Table 9:** Corpus associated with concatenation 3 and obtained from a control subject.

Locutor	ε	Enunciation
Exp	10	why do we use calendars?
HC	11	[noise is heard in the room] well... why what?
Exp	12	why do we use calendars?
HC	13	well to know what day it is
Exp	14	mhm
HC	15	and…
Exp	16	ok

All speech acts are represented.

In every other concatenation, additional elaborations were given prior to the experimenter’s validation of the correct answer, indicating a high convergence among the participants (Figure 1, Left). Subsequent tests of correlation between vergence and celerity measurements were performed given all concatenations and for both subjects. Vergence and celerity were found be strongly correlated over the Shwachman-Diamond subject [*r_(7)_* = 0.87, *p* < 0.001]; but not in the control subject [*r_(7)_* = 0.008, *p* = 0.98] suggesting that celerity is limited by vergence (i.e., cooperation) only in the SDS patient.

#### 4.2.3 Strategic management of dialogue: drifting the experimenter outside the framework of the ongoing task

The sequence is particularly interesting as it also demonstrates how CG strategically utilized the dialogic system to expedite the completion of the ongoing task. From the outset, CG’s task and interlocutor alignment shifted beyond the divergence threshold at *ε* = 5 (Figure 1, Bottom). Although alignment toward the experimenter occasionally returned to the convergence threshold at *ε* = 14, task alignment never crossed this threshold again, indicating a cooperative deficit in resolving the task at hand. Moreover, CG managed to involve the experimenter in this divergence; given the experimenter’s role in fostering discourse and listening, they consistently sought to maintain alignment with CG, evidenced by the experimenter’s vergence toward CG remaining nearly constant at *V* = 1, except when attempting to refocus on the task. This dialogic link, and thus the convergence between the interlocutors, was maintained at the expense of the experimenter’s task alignment, which crossed the divergence threshold at *ε* = 21 during the second concatenation and never returned to convergence, despite multiple attempts, as indicated by subtle decreases in the experimenter’s vergence toward CG (Figure 1, Bottom).

In the dialogic game that unfolded, the experimenter’s intrinsic context notably included a time constraint (the time available for administering tests to the subjects was severely limited due to a train schedule). Consequently, it can be presumed that during this sequence, he endeavored to direct the subject’s focus toward completing the task as quickly as possible. However, he failed in this effort, getting sidetracked by the patient’s digressions in an attempt to maintain the dialogic connection, which ultimately led to the premature termination of the task and the failure of the subject to effectively perform it.

#### 4.2.4 The effect of prompting: empirical evidence of the divergence between interlocutors’ goals

In the section, we will focus on a brief section of this dialogue. The 7th concatenation (i.e., item 7) was heavily laden with the patient’s personal anecdotes, resulting in minimal convergence (Figure 1, Left). During this sequence, a prompt was given to the patient, noting the experimenter’s urgency and the need to quicken the pace by refocusing the interaction on task completion (Figure 1, Right, *ε* = 302) (Table 10).

**Table 10:** Speech act produced at *ε* = 302 (Reminder on the Figure 2).

Locutor	*ε*	Enunciation
Exp	302	We really need to finish now because I’m gonna take the train.

Ordinarily, this statement should have elicited a particular effect on the dialogic system due to its form, which could be construed as a reprimand, and the final justification intended to enhance the patient’s cooperativeness.

**Figure 2:**
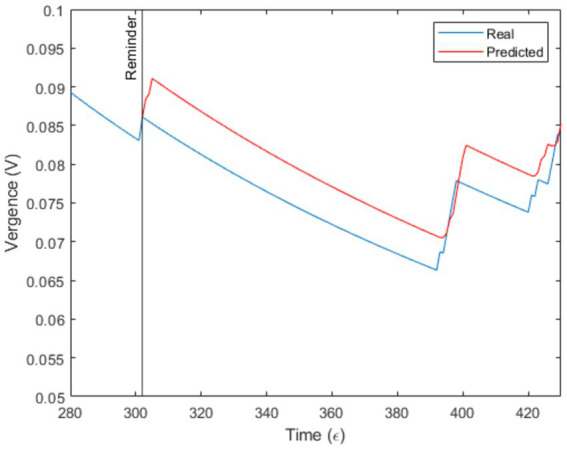
Prediction of the effect of reminder by a trained Recurrent Neural Network on the vergence values of the first 302 speech acts.

To test this claim computationally, a Recurrent Neural Network (RNN) was specifically trained on this interaction to predict convergence in the participants’ exchange over time. The neural network was trained using the dynamics of this single interaction from the initial speech act (*ε* = 1) to the corrective reminder (*ε* = 302), and was then tasked with forecasting the continuation of the interaction, adjusting every three speech acts (Figure 2).

Figure 2 displays the forecasting of the dynamics of the sole dialogue used to train the model, after the reminder induction (i.e., following a positively polarized speech act). Prediction suggests a divergence in the interlocutors’ objectives.

In a human context, one might assume that a reminder would refocus the subject to the task by increasing vergence, as attested by computational modeling which yet relies solely on past interactive dynamics of this specific dialogue and does not account for social norms or prosody (Figure 2, red curve). However, reminder had no particular effect on vergence (Figure 2, blue curve), which continued to decrease significantly, indicating a substantial divergence in the objectives pursued by the speakers and suggesting a lack of cooperation from the patient.

#### 4.2.5 Cooperation and interactive engagement: the effect of cost in a case study

**Figure 3:**
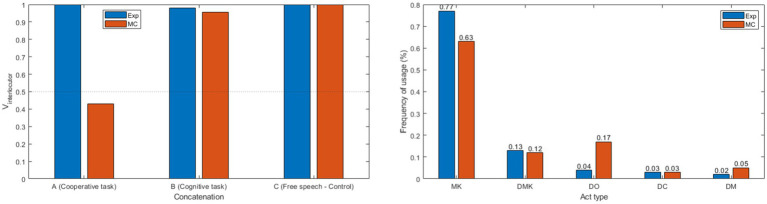
Pragmatic and Kinetic Analysis of Experimenter-MC Interaction in 2TK. The top-left figure illustrates the vergence values toward the interlocutor in three scenarios: cooperative task (A: basket); cognitive task (B: “Picture Span” subtest from WISC-V); and free dialogue (C). The top-right figure shows the frequency of speech act usage for each interlocutor [MK, Make-Know; DMK, Do Make-Know; DO, Do; DC, Do Can; DM, Do Must].

The second unitary case we present, MC, exemplifies the challenges faced by caregivers and educators of SDS in cooperative interactions with unequal benefit/cost ratios between participants. This case also provides a detailed illustration of the impact of SDS’s difficulties in integrating contextual elements to produce appropriate responses. Generally, all observed aspects can be explained by economic parameters in terms of cognitive and motor costs. Figure 3 presents the results from three interaction sections: a cooperative task (the basket, cognitively undemanding and requiring active motor cooperation); a cognitive task (the “Picture Span” subtest, cognitively demanding but not requiring cooperation); and a free dialogue situation (i.e., control situation, cognitively undemanding).

#### 4.2.6 The economy of reactive action and the avoidance of cooperative engagement

Analysis regarding the frequency of usage of speech act types for each interlocutor is presented in Figure 3 (Top-Right). Data suggested distinguishable usage rates between the two interlocutors.

Generally, it is observed that both interlocutors asked the same number of questions (DMK%: Exp = 0.13; MC = 0.12) and produced the same rate of requests formulated as an invitation to cooperate (DC%: Exp = 0.03; MC = 0.03). Interestingly, the profiles begin to diverge in terms of speech acts that facilitate the development of the discursive situation, namely MK and DO, which share the same properties but differ in terms of motor action and verbal production. Notably, the experimenter produces more information verbally (MK%: Exp = 0.77) than through motor action (DO%: Exp = 0.04), in contrast to the SDS patient who produces, proportionally, more information through motor action (DO%: MC = 0.17) than verbally (MK%: MC = 0.63). Interestingly, when the usage rates of these two types of acts are combined, similar frequencies are observed between the two interlocutors (DO% + MK%: Exp = 0.81; MC = 0.8) (Figure 3, Right).

The observed variance in the frequency of elaborative act usage prompts an inquiry into its underlying reasons. A plausible explanation could be the cognitive exertion involved in response generation. The ease of resorting to actions or gestures, as opposed to crafting a contextually apt and pertinent response, is evident. This conjecture is bolstered by the observation of the interactive space occupation and convergence (Figure 3, Left; Table 11). A retrospective analysis of the corpus reveals a predominance of reactive sequences from the patient, often manifested through laughter or sighs. An illustrative example is a drawing activity initiated by the subject for taking a break between the two battery administrations (Table 11).

**Table 11:** Example of a corpus where MC uses reactive actions to reduce the cost of interaction, but cooperation remains minimal.

Locutor	*ε*	Enunciation
Exp	127	well I’m not bad?
MC	128	huh?
Exp	129	I’m not bad?
MC	130	[laughs]
Exp	131	no I’m bad?
MC	132	huh…
Exp	133	I do not know, do they normally have shoulder pads?
Exp	134	they are a little big there but...
MC	135	[laughs]

This segment of the corpus focuses on the experimenter’s pursuit of the patient’s evaluation of their drawing. A notable disparity is immediately evident between the interlocutors, despite their shared task, as the subject struggles to grasp the request for evaluation. This is unexpected, given the subject’s satisfactory performance in theory of mind tasks involving comparable levels of inference. Two significant observations emerge from this scenario: the request for evaluation remains persistently unfulfilled, and there is a lack of substantial progress toward the interlocutor’s objective, despite their repeated attempts to engage the subject. Thus, once again, the difficulties in obtaining an effective cooperative interaction with patients suffering from Shwachman-Diamond syndrome are observed; particularly in the necessity to repeat inductions to obtain verbal information rather than gestural.

#### 4.2.7 Social disinhibition and directivity

The analysis of the TEST-ASAP subtasks, notably the ‘Syllogism’ task discussed later in the manuscript, has revealed preliminary indications of a distinct form of social disinhibition. This is characterized by the violation of established societal norms in the pursuit of perceived benefits (Table 12).

**Table 12:** Example of a section where the subject makes inappropriate use of Do-Must speech act (*ε* = 366).

Locutor	*ε*	Enunciation
Exp	362	well, we are done for the morning
Exp	363	cool
Exp	364	we were quick
Exp	365	it’s good
MC	366	**you draw a footballer now**
Exp	367	ah you want to make me suffer in fact
MC	368	yeah

Bold terms indicate the inappropriate use of a “Do-Must” speech act.

In the case of patient MC, our prior analysis has revealed a distinguishable frequency of acts producing information in comparison to the interlocutor (Figure 3, Right). Notably, this patient’s interactions also exhibit an over-representation of “Do Must” speech acts, signifying direct orders devoid of alternative options (DM%: Exp = 0.02; MC = 0.05). This tendency is particularly striking when considering the context of WISC-V administration, where the experimenter’s use of such acts is prominent. Consequently, it appears that patient MC may not be fully conforming to the typical social norms that govern interactions between healthcare professionals and young patients, characterized by a substantial degree of asymmetry.

#### 4.2.8 The impact of individual economic value on cooperative behavior

Results regarding the effects of personal economic value in cooperation are presented in Figure 3 (Left) and Tables 13–15. The data suggested a strong impact of the interactive context on the convergence between the interlocutors.

**Table 13:** An example of interaction obtained in a free dialogue situation.

Locutor	*ε*	Enunciation
MC	6	Are you... do you work here?
Exp	7	not at all
Exp	8	I do not even live here
Exp	9	I drove 400 km this morning to come
MC	10	oh
Exp	162	I do not even know...[we hear someone playing guitar and singing in the hallway] f*** is he still here?
MC	163	[laughs]
Exp	164	you will not tell your mother I said bad words
MC	165	[laughs]
MC	166	yes
MC	167	[laughs]
MC	168	you are so funny frankly
Exp	168	ah thank you it’s cool

The concatenation involved a guitar player present in the hospital corridor during the evaluation. It is discontinuous as the concatenation was reopened each time the musician was heard. The excerpt includes 13 speech acts out of the total 37 in the concatenation.

**Table 14:** Example of interaction obtained in a cognitive task situation.

Locutor	*ε*	Enunciation
Exp	286	look it right, ok?
MC	287	yeah
Exp	288	ok good
Exp	289	ok
MC	290	all that boring stuff there
Exp	291	yeah
MC	292	ok nice
Exp	293	planet shoe alarm clock star planet shoe alarm clock star ok
MC	294	planet shoe alarm clock star ok
MC	295	nice
MC	296	hourglass... heart… cube
MC	297	hourglass kite cube heart
Exp	298	ok nice

The concatenation concerned the performance of the WISC-V “Picture Span” subtest. It is contained since the concatenation was not reopened after its closure and the example concerns 13 speech acts out of the 101 total of the concatenation.

**Table 15:** Interaction obtained in the cooperative task situation.

Locutor	*ε*	Enunciation
MC	65	do you have any colored pencils?
Exp	66	colored pencils?
Exp	67	yeah I do
MC	68	[MC sings]
Exp	69	but the lead is not in a good state
Exp	70	so I’ll give you a pencil sharpener
Exp	71	but you need the basket to put the cuttings
MC	72	it’s okay
MC	73	[MC presses hard on the pencil to get the lead]
MC	76	[MC sings]
MC	77	it does not color the way I want
Exp	78	how do you want to color?
MC	79	the shield
Exp	80	[laughs]
MC	123	well I did not color like I wanted because it wasn’t cut
Exp	124	well prune it
MC	125	oh my knight is so cool
MC	643	draw the pants
MC	644	I want to see the pants
Exp	645	could you get me the basket then?
MC	646	huh?
Exp	647	could you get the basket, by any chance?
MC	648	which basket?
Exp	649	to uh put the cuttings
Exp	650	so I can color it
MC	651	What’s Nike looks like again?

This discontinuous concatenation involved the pause time where the interlocutors engaged in a drawing activity upon the patient’s request. The complete set of 26 speech acts from the concatenation are presented.

In this example of an interaction obtained in a free dialogue situation (Table 13, cognitively non-costly), a strong convergence between the interlocutors can be observed (Figure 3, Left, Condition “C”; *V_int_*: Exp = 1; MC = 1).

In this second example, which depicts a section of interaction obtained during a cognitive task (Table 14), a strong convergence between the interlocutors is also observable (Figure 3, Left, Condition “B”; *V_int_*: Exp = 0.97; MC = 0.98).

Finally, the most noteworthy example is the interaction observed in a cooperative task setting, which significantly contrasts with the measurements obtained in other situations (Table 15).

A notable differentiation in the vergence toward the interlocutor between the two participants emerges (Figure 3, Left, Condition “A”; *V_int_*: Exp = 0.1; MC = 0.4). In this scenario, the convergence between the interlocutors is remarkably weak, causing the patient’s measured values to drop below the divergence threshold (*V* < 0.5). This underscores that less than half of the patient’s speech acts facilitate the resolution of the concatenation.

From these three observation points (Figure 3, Left), it can be concluded that the interactive engagement of this patient was dependent on two critical parameters: the estimated cost of producing the interactive sequence, relative to the expected benefits; and the interactive context of the exchange, particularly in terms of the directivity of the anticipated benefits.

### 4.3 Group study

**Figure 4:**
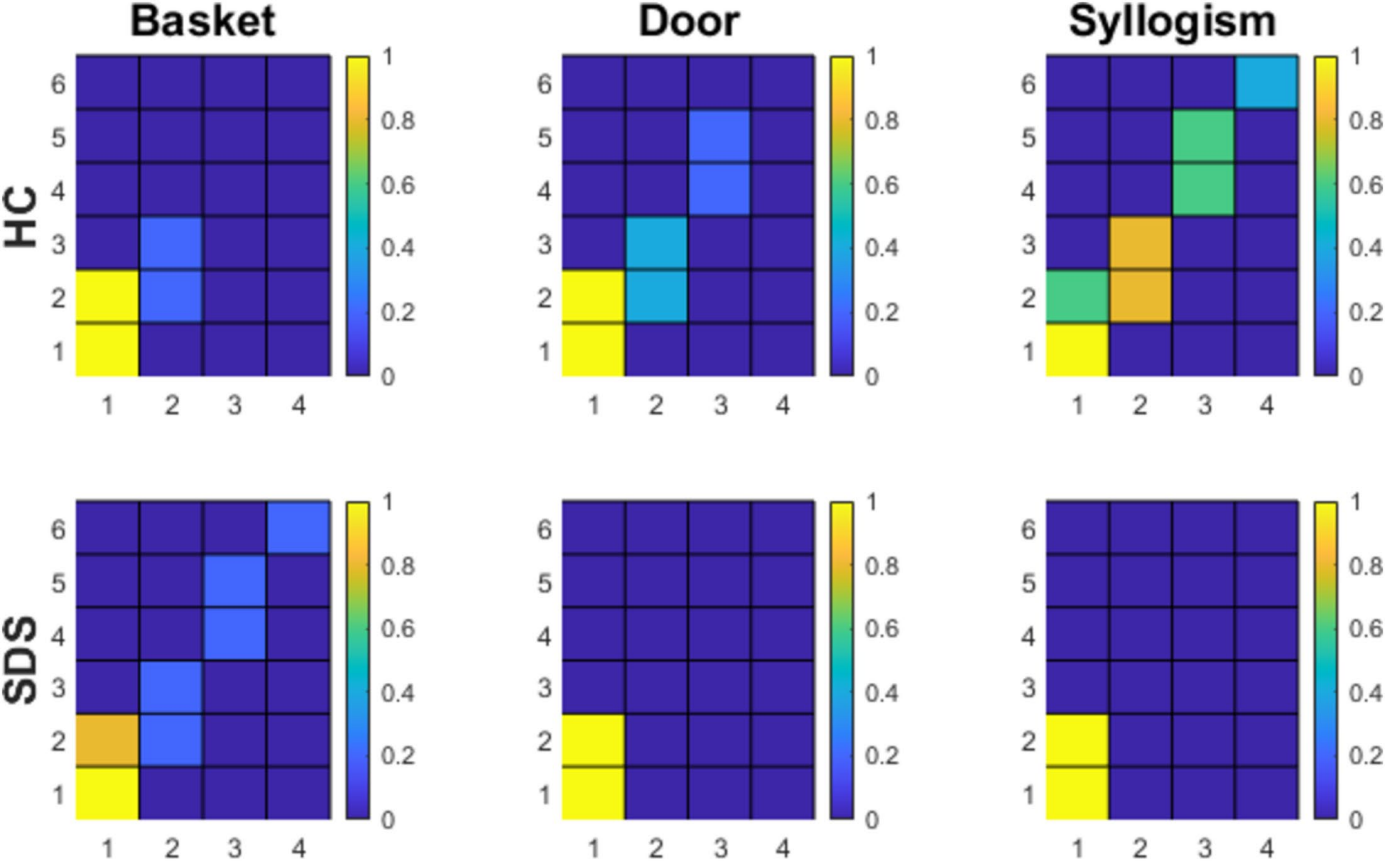
Results from the TEST-Asap for both groups, projected onto matrices in 2TK. Each cell of the matrix represents a topological position, and the associated color gradient corresponds to the probability of passing through this position at least once during the interaction.

The outcomes pertaining to the topological-kinetic measurements of the TEST-ASAP for both groups are presented in Figure 4. The data revealed distinguishable behaviors between the two groups, with more homogeneous behaviors observed in the SDS group.

In the ‘Basket’ subtask, both groups displayed differentiable performances. Four of five control subjects immediately responded to the indirect speech act to retrieve the basket, whereas one asked for the instruction to be repeated due to inattention. The SDS group showed comparable responses, except for one male patient who outright refused to execute the task, even after a direct prompt, indicating a notable unwillingness to cooperate.

Regarding the “Basket” subtask, the groups exhibited distinguishable performances. In the control condition, although 20% of participants did not immediately respond to the direct request—as indicated by occupying position (*x* = 2, *y* = 2), corresponding to no response, and position (*x* = 2, *y* = 3), corresponding to an indirect reformulation of the request—all participants eventually converged to position (*x* = 1, *y* = 2), representing the completion of the dialogic task and thus the retrieval of the basket. In contrast, within the SDS group, while 80% of participants also converged to position (*x* = 1, *y* = 2), one subject outright refused to cooperate. This refusal was marked by a progression through successive positions, ultimately reaching position (*x* = 4, *y* = 6), which corresponds to the failure of the cooperative task.

Similarly, the “Door” subtask revealed notable contrasts between groups. In the SDS group, 100% of participants directly converged to position (*x* = 1, *y* = 2), representing the successful completion of the dialogic task. However, in the control group, 40% required an indirect reformulation of the request, as indicated by occupying positions (*x* = 2, *y* = 2) and (*x* = 2, *y* = 3). Furthermore, one control subject required a direct reformulation of the task, evidenced by their request for clarification at position (*x* = 3, *y* = 4) (“Should I close the door?”) and the experimenter’s subsequent direct reformulation at position (*x* = 3, *y* = 5; “Yes”). This sequence ultimately led the group to converge at position (*x* = 1, *y* = 2), indicating task success and the act of closing the door.

Finally, the “Syllogism” subtask revealed entirely contrasting patterns. Participants in the SDS group immediately stood up and went to fetch their parents to eat as soon as the task concluded, directly converging at position (*x* = 1, *y* = 2), representing task completion and the child’s departure. In contrast, only 60% of control group participants reached this position during the task. Among the control group, 80% required at least one indirect reformulation of the request, as indicated by positions (*x* = 2, *y* = 2) and (*x* = 2, *y* = 3); 60% required a direct reformulation, occupying positions (*x* = 3, *y* = 4) and (*x* = 3, *y* = 5). Finally, one of the control group converged at position (*x* = 4, *y* = 6), corresponding to task failure. This specific control child remained seated until the experimenter escorted them back to the waiting area.

The TEST-ASAP’s triple dissociation thus offers insights into the granularity of SDS cooperation. The “Basket” subtask assesses cooperative propensity when the procedure is explicitly clear, yet not necessary for achieving the associated goal of using a poorly sharpened pencil. Here, the patient gains no direct benefit from cooperation beyond fulfilling the experimenter’s request. In contrast, the “Door” subtask examines cooperation propensity under an implicit procedure requiring minimal inference to interpret indirect actions (e.g., addressing a draft), which benefits both parties and aligns with social norms (e.g., closing a door when with a healthcare professional). The “Syllogism” subtask evaluates cooperation when the procedure is explicit and benefits primarily the patient (e.g., taking a break to eat and relax). However, this test also entails violating a social rule (i.e., not rising without the healthcare professional’s permission), as the immediate satisfaction of speech act conclusions derived from presupposed premises demands. Analysis of these three tests suggests that SDS patient behaviors are spectrum-based and context-dependent, particularly when benefits to the patient are minimal. As such, cooperation propensity increases with anticipated personal benefits. In contrast, performance, does not seem influenced by the level of indirectness, suggesting it hinges solely on the economic aspects of interaction. This is also suggested from the greater perceived value of personal benefits over prevailing social norms, as indicated by the observed lifting of social inhibition in the “Syllogism” subtest.

## 5 Discussion

Utilizing computational modeling methods, this study has provided significant insights into the pragmatic and sociocognitive profile of children with Shwachman-Diamond Syndrome. It has also confirmed certain ubiquitous cooperative behaviors they exhibit, potentially establishing these as core symptoms of the clinical condition.

Moreover, our findings align with parental reports that suggest a unique “economic mechanic” in the social cooperation of children with SDS, examining how these behaviors differ from those of neurotypical children in dialogical-pragmatic tasks.

Our analyses spanned several contexts, from ecological settings with varying cost–benefit balances to controlled cognitive tasks, each illustrating a distinct pragmatic profile in Shwachman-Diamond patients. Notably, Shwachman-Diamond children, while displaying a robust sensitivity to indirect speech acts, seem governed by an economic principle of interaction where behaviors were predominantly influenced by their direct personal benefits, even at the expense of social norms they understood.

For example, during the administration of the “Comprehension” subtest of the WISC-V, one patient, expressing fatigue, significantly derailed the task by digressing to personal anecdotes, illustrating how Shwachman-Diamond patients may disregard task objectives in favor of pursuing personal goal. Similarly, in a cooperative interaction involving no apparent benefit to the patient, another Shwachman-Diamond child implicitly and explicitly refused to cooperate selectively in task which did not present direct benefit from him.

These findings suggest that while Shwachman-Diamond children may comprehend social cues and norms, their interaction strategies are primarily shaped by perceived personal gains, challenging the assumption that cooperative engagement are universal drivers in social interactions (Grice, 1975).

Their profile is thus distinguishable from other pathologies that exhibit deficits in theory of mind and cooperativity, notably Autism Spectrum Disorders (ASD), where available data in the literature suggest that their inclination to communicate depends on the directiveness of the request itself (i.e., patients cooperate if the request is formulated in a direct, rather than implicit, manner; Deliens et al., 2018; Gerardin-Collet, 1999; Kissine et al., 2016).

Interestingly, the behavioral parameters measured at the dialogic level using the 2TK computational modeling seem to closely align with the data obtained from neuroimaging studies. Indeed, the anterior cingulate cortex, the left inferior frontal gyrus, and the precuneus, whose anatomy appears to be altered in Shwachman-Diamond Syndrome, are two key regions enabling sensitivity to speech acts. This is particularly true for identifying pragmatic elements carried by indirect speech acts, as well as for maintaining a coherent representation of discourse and mentalization (Bašnáková et al., 2014; Feng et al., 2017, p. 201; Licea-Haquet et al., 2019; Reyes-Aguilar et al., 2018; Senft, 2014; Shibata et al., 2011; van Ackeren et al., 2012). Consistent observations have also been noted with orbital frontal involvement. Indeed, some experimental studies suggest that the orbitofrontal cortex is a key region of neural decision-making systems, characterized by strong activation during reciprocal social interactions in contrast to purely monetary rewards (Rilling et al., 2002). Thus, the observed behavioral tendencies in children with Shwachman-Diamond syndrome to prioritize direct personal benefits over cooperative social interactions could reflect an altered valuation system where typical social rewards do not elicit the expected neural responses due to an atypical functioning of the anterior cingulate, orbitofrontal cortex and related structures.

The behaviors centered around personal gains observed in children with Shwachman-Diamond Syndrome carry important implications in the context of legal and social frameworks, particularly as these behaviors may persist into adulthood. Recent research into legal implications for individuals with intellectual disabilities provides a valuable framework for understanding how the behaviors observed in Shwachman-Diamond Syndrome might intersect with legal systems. Giannouli’s comprehensive review (Giannouli, 2016) highlights that cognitive and social impairments often place individuals with intellectual disabilities at a disadvantage in both civil and criminal domains, particularly in contexts where societal expectations of cooperation and implicit norm adherence conflict with individual capacities. Key challenges include difficulties in understanding contracts, navigating consent, and managing financial obligations, often exacerbated by a focus on immediate rewards and reduced cognitive flexibility. In SDS, these vulnerabilities may be compounded by a pragmatic preference for actions yielding direct personal benefits, even when they conflict with social norms.

While individuals with SDS are not overrepresented in criminal justice systems, as noted in studies of broader intellectual disabilities populations (King and Murphy, 2014), their unique behavioral tendencies may increase the risk of legal misinterpretations. For example, diminished social inhibition or opportunistic interactions could be perceived as noncompliance in structured legal environments, particularly when cooperative norms are presumed. Giannouli also notes the influence of societal bias and institutional histories in amplifying legal vulnerabilities, a factor that may similarly affect SDS patients through patterns of misunderstanding or marginalization (Giannouli, 2016).

Moreover, emphasis on personal economic gain observed in SDS behaviors mirrors broader findings on social rejection and legal challenges for individuals with disabilities (Wright, 2016), where misaligned expectations can lead to conflicts in educational and social settings, which thus may escalate in legal contexts.

Future investigations could benefit from larger, more diverse cohorts to enhance the generalizability of findings and better capture interindividual variability in SDS. Additionally, comparative studies with other neurodevelopmental conditions, such as Autism Spectrum Disorders or Attention Deficit/Hyperactivity Disorder, could help delineate specific versus shared pragmatic features. Integrating behavioral data with neuroimaging techniques would further elucidate the neural mechanisms underpinning the observed behaviors. Longitudinal studies are also warranted to assess the developmental trajectory of these pragmatic behaviors and their impact on social functioning. Finally, these findings could inform the design of targeted interventions, such as tailored communication strategies or educational programs, to enhance cooperation and social integration in SDS patients.

Finally, the legal challenges associated with SDS highlight the need for interdisciplinary approaches that bridge neuropsychology, law, and social policy. By addressing the specific interaction strategies of SDS individuals, legal frameworks can better accommodate their unique profiles, reducing systemic vulnerabilities and promoting equitable outcomes. Future research should focus on longitudinal studies to examine the legal trajectories of SDS individuals and the efficacy of neuropsychological-informed interventions in mitigating their risks within legal systems.

However, several limitations should be acknowledged for this study. The small sample size limits the statistical power and the generalizability of results. Additionally, while the controlled experimental setting ensures the precision of data collection, it may not fully capture the dynamics of real-life interactions. Furthermore, potential biases arising from the hierarchical relationship between experimenters and participants must be considered. Lastly, while this study emphasizes computational and neuropsychological measures, incorporating qualitative data, such as parental reports or ethnographic observations, could provide a richer understanding of SDS social behaviors. Addressing these limitations in future research could strengthen the validity and applicability of results in clinical and educational contexts.

## Data Availability

The raw data supporting the conclusions of this article will be made available by the authors, without undue reservation.
