# Supplementary material for: Self-beneficial transactional social dynamics for cooperation in Shwachman-Diamond syndrome: a mixed-subject analysis using computational pragmatics
Source: Front Psychol. 2025 Jan 22;15:1459549. doi: 10.3389/fpsyg.2024.1459549 (PMC11794258; doi:10.3389/fpsyg.2024.1459549)
Supplement: Supplementary file 1 [file Data_Sheet_1.pdf]

1 Definition and properties of the 2TK model

1.1 The interaction function

The interaction function is the foundational formula of the 2TK model. It reports the state of the dialogical system as a function of dialogical time (i.e. the statement ; the speech act ; or the indexical, depending on the resolution).

$$\Psi(\xi, \epsilon, \sigma_n) \tag{1}$$

With :

- ξ as the position of the statement in the dialogue depending on x and y ;
- ϵ as the dialogical time (which is iterative and relative to the structure in which it is measured and which belongs to σ) ;
- σ_n as a set of parameters that describe the system (e.g. its metric, rhetorical relation, speech act, the state of the dialogue etc....) and which depends on x, y, and ϵ.

1.1.1 Basic definitions and properties

The following definitions represent values that the sigma parameters can take.

Definition 1 (Dialogue) *The dialogue Λ represents the unit of macroscopic analysis and is defined by the set of successive planes occuring in the dialogue.*

$$\Lambda = \sum_{i=1}^{n} \Pi_i \tag{2}$$

With :

- Π_i *as the successive dialogical plans occuring in the dialogue.*

Definition 2 (Plans) *The plan of dialogue Π is the sum of the concatenations $\mathfrak{C}$.*

$$\Pi = \sum_{i=1}^{m} \mathfrak{C}_i \tag{3}$$

$$\tag{4}$$

With :

- $\mathfrak{C}_i$ *as the concatenation ;*

Definition 3 (Concatenation) *The concatenation* $\mathfrak{C}$ *is defined by the set of speech acts* κ*.*

$$\mathfrak{C} = \sum_{i=1}^{l} \kappa_i \tag{5}$$

Where :

- κ_i *is the speech act;*

Definition 4 (Speech act) *The speech act* κ *is defined by its information, and its type* k*.*

$$\kappa = (q, k) \tag{6}$$

Where :

- q *is the information considered for the speech act. It can represent a character string, a quantity of information measured in bits, keywords (which can be keywords present in the sequence as formulated by an interlocutor or keywords from perlocutionary description systems). In order to simplify the construction of models, it is possible to approximate speech act by the reduction of its measure to its type as* $\kappa \approx (k)$*.*

- k *is the type.*

Property 4.1 (Polarity) *The polarity* δ *of a speech act* κ *is defined by its provability within the dialogical structure to which it is compared.*

$$\delta = \begin{cases} 1 \Leftrightarrow \kappa(\epsilon_{n+i}) \vdash \kappa(\epsilon_n) \\ 0 \Leftrightarrow \neg(\kappa(\epsilon_{n+i}) \vdash \kappa(\epsilon_n)) \end{cases} \tag{7}$$

Definition 5 (Act type) *The act type* k *is defined by the grammatical form*

of the statement.

$$k = \begin{cases} DO \\ MK \\ DMK \\ DC \\ DM \end{cases} \tag{8}$$

With:

Act (2TK)	Act (Caelen, 2003)	Searle equivalent (Searle, 1969)	Meaning (Caelen, 2003)
DO	F^{A}	Declarative	Do or carry out an action (in verbal or non verbal)
MK	F^{S}	Assertive or expressive	(make-known) communicate an information
DMK	F^{FS}	Directive	(do make-known) ask for an information
DC	F^{P}	Promissive with delegation	(do can) give a choice, make an invite
DM	F^{D}	Directive	(do must) oblige without giving an alternative

Table 1: Characterization of speech acts in the 2TK model and relationship with their source models.

Definition 6 (Address) *The address ξ is defined by its topological coordinates x and y.*

$$\xi = (x, y) \tag{9}$$

Definition 7 (State of dialogue) *The state of dialogue is a stable state containing the measures describing the system and that is only transformed by the next speech act.*

$$D_i = D_{i+1} \Leftrightarrow \kappa_{i+1} \tag{10}$$

Property 7.1 (State of dialogue transition) *Any speech act κ formulated by one of the speakers implies the transformation of the the state of dialogue D_i to its next state D_{i+1} which will be a function of the type of speech act k, its direction, its polarity δ, and the rhetorical relation ρ associated with the previous address ξ_{n-1}.*

$$D_i + \kappa_{i+1} \xrightarrow[k_B^A,\ \delta]{\rho} D_{i+1} \tag{11}$$

1.1.2 State of the dialogical system : overview of some possible values for the internal parameters σ

Definition 8 (The Vergence (V)) *The vergence is a measure of interlocutor convergence to an object represented by the ratio between the number of positively polarized speech acts present in the dialogue or in the section of dialogue and the total number of speech acts. It is defined as follow :*

$$V = \frac{\sum \kappa_{i,\delta(1)}}{\sum \kappa_i} \tag{12}$$

Property 8.1 (The maximal convergence) *The maximal convergence of the dialogue or the section of dialogue is achieved if and only if all speech acts present in the dialogue or in the section of dialogue are positively polarized.*

$$V = V(Max) \Longleftrightarrow V = 1 \tag{13}$$

Property 8.2 (The convergence) *In a dialogue or a dialogue section, there is convergence between the interacting elements if and only if* $V > 0.5$.

Reciprocal 8.1 (The divergence) *In a dialogue or a dialogue section, there is divergence between the interacting elements if and only if* $V \leq 0.5$.

Definition 9 (Celerity (C)) *The Celerity of a dialogue or a section of dialogue is the ratio between the number of speech acts needed to solve a goal and the number of speech acts produced to solve the goal. The example used corresponds to a concatenation. It is defined as follow :*

$$C = \frac{\sum \kappa_{i,d_{(1)}}}{\sum \kappa_i} \tag{14}$$

Theorem 1 (Primacy of Celerity Theorem) *For a given dialogue, the vergence* V *cannot exceed the celerity* C *:*

$$V \leq C \tag{15}$$
